# Effects of *Bacillus licheniformis* 809 and *Bacillus subtilis* 810 supplementation on in situ degradation of a high-concentrate byproduct-based diet in forage-fed beef cows

**DOI:** 10.1093/tas/txag055

**Published:** 2026-05-02

**Authors:** Arnaldo C Limede, Rodrigo S Marques, Fernando A A Cidrini, Erollykens F Santos, Paolo Fantinati, Bruno I Cappellozza

**Affiliations:** School of Animal Sciences, Virginia Polytechnic Institute and State University, Blacksburg, VA, 24061, United States; School of Animal Sciences, Virginia Polytechnic Institute and State University, Blacksburg, VA, 24061, United States; School of Animal Sciences, Virginia Polytechnic Institute and State University, Blacksburg, VA, 24061, United States; School of Animal Sciences, Virginia Polytechnic Institute and State University, Blacksburg, VA, 24061, United States; Novonesis, Parma, 43126, Italy; Novonesis, Lyngby, 2800, Denmark

**Keywords:** *Bacillus* spp, direct-fed microbial, *in situ*, nutrient disappearance

## Abstract

This study evaluated the effects of *Bacillus*-based direct-fed microbial (**DFM**) supplementation on in situ nutrient disappearance and degradability of high-concentrate diets. Fifteen rumen-cannulated cows were used in a completely randomized design. Treatments consisted of a forage-based diet plus 500 g of protein-mineral (390 g dried distillers grain + 110 g vitamin-mineral mix; as-fed basis) supplement mixed with **1)** 3 g (as-fed basis) of a *Bacillus*-based DFM containing *Bacillus licheniformis* 809 and *B. subtilis* 810 (**BAC**; *n* = 7; 2.2 × 10^9^ CFU of the mixture/g; Bovacillus, Novonesis, Lyngby, Denmark), or **2)** without BAC (**CON**; *n* = 8). Samples of 4 high-concentrate, byproduct-based diets (A, B, C, and D) were collected from a commercial feedlot in South Africa. These diets were composed of hominy chop, corn silage, *Eragrostis* hay, cottonseed cake, molasses syrup, urea, limestone, salt, and a mineral-vitamin premix, and subsequently dried for analysis. From days 28 to 32, each diet was placed in Dacron bags and introduced through the cannulas. Residuals were dried, and DM, NDF, and ADF content were determined. No treatment × hour interactions were detected (*P* ≥ 0.11) for nutrient disappearance in any of the diets. Dry matter and NDF disappearance were greater (*P* ≤ 0.05) in diets A, C, and D for cows fed BAC compared with CON, whereas no differences were observed in nutrient disappearance from diet B (*P* ≥ 0.33). Similarly, ADF disappearance was greater in diet A (*P* = 0.04) and tended to be greater in diet C (*P* = 0.07) in BAC vs. CON and was unaffected by treatment in diets B and D (*P* ≥ 0.13). Combining all four diets, degradation of fraction B (degradable fraction; total pool disappearing at a measurable rate) of DM was greater for BAC vs. CON (*P* = 0.02), whereas degradation of fraction C (undegradable fraction; total unavailable pool in the rumen) tended to be lower for BAC than CON (*P* = 0.08). Evaluating NDF degradation across diets, the degradation of fraction B tended to be greater for BAC vs. CON (*P* = 0.10), whereas effective degradability was greater for BAC compared with CON (*P* = 0.02). No treatment effects were observed on NDF fraction C (*P* = 0.18) or degradation rates (% per h; *P* = 0.92) between treatments. Collectively, feeding a *Bacillus-*based DFM improved in situ disappearance and the potentially degradable nutrient pool of a high-concentrate byproduct diet in forage-fed beef cows.

## Teaser

Adding a *Bacillus*-based direct-fed microbial to a forage-based diet in beef cows enhanced ruminal disappearance and degradability of nutrients from a high-concentrate byproduct diet. These results underscore the enzymatic potential of *Bacillus* spp. to improve nutrient utilization even across contrasting diet types.

## Introduction

Direct-fed microbials (**DFM**), particularly those based on *Bacillus spp*., have gained attention for their enzymatic potential to enhance nutrient utilization in the rumen and lower digestive tract (Cappellozza et al. 2025). In the companion study (Limede et al. 2025), supplementation with *Bacillus licheniformis* 809 and *B. subtilis* 810 (**BAC**) improved voluntary forage DMI, in situ disappearance of forage DM and NDF, and total-tract DM, CP, NDF, and ADF digestibility in rumen-cannulated beef cows fed a low-quality mixed grass hay, without significantly altering the ruminal bacterial community or fermentation profile. These effects were likely driven by the ability of *Bacillus spp.* to adhere to feed particles and secrete enzymes that aid in the breakdown of plant cell wall components (Schallmey et al. 2004; Luise et al. 2022). *Bacillus spp.* have also gained attention as effective DFM because they remain stable under a wide range of feeding strategies (Luise et al. 2022; Cappellozza et al. 2023a), can modulate digestion throughout the gastrointestinal tract (Green et al. 1999; Fuerniss et al. 2022), help support the microbial community (Segura et al. 2020), and enhance intestinal barrier integrity and function (Rhayat et al. 2019; Boll et al. 2024).

Bacillus-based DFM are increasingly incorporated into feedlot and byproduct-based finishing systems, yet their mechanisms of action within the ru men remain insufficiently defined. Given that, *Bacillus spp.* can affect ruminal processes through extracellular enzyme production, metabolite secretion, and interactions with feed substrates, some of which may occur independently of major shifts in the ruminal microbiome (Schallmey et al. 2004; Luise et al. 2022). Therefore, evaluating their activity within a stable rumen environment is an important step for defining their functional potential. Using a consistent forage baseline allows isolation of the microbial activity attributable to the DFM, providing a platform for assessing effects on the degradation of high-concentrate byproduct feedstuffs. Additionally, in situ degradation provides an indicator of the extent to which feed components are broken down in the rumen and offers insight into potential in vivo nutrient availability (Foster et al. 2023). Previous work has shown that *Bacillus*-based DFM can alter fermentation patterns and feed efficiency in cattle fed high-concentrate diets (Dias et al. 2022; Cordeiro et al. 2024). However, because diet composition strongly influences ruminal microbial function and nutrient degradation dynamics (NASEM 2016), it is not yet clear whether enzyme activity from *Bacillus-*based DFM can enhance in situ disappearance and degradation of high-concentrate substrates when cattle consume a forage-based basal diet. The 4 diets selected for this trial represent typical byproduct-based transition and finishing rations used in commercial feedlots in South Africa. Given that, we hypothesized that supplementing *Bacillus licheniformis* 809 and *Bacillus subtilis* 810 in a forage-based basal diet would enhance the in situ ruminal disappearance and degradation of DM and fiber fractions of these high-concentrate byproduct feeds. Including these diets allowed us to evaluate DFM effects under practical, regionally relevant high-concentrate feeding conditions. Therefore, the objective of this study was to evaluate the effects of supplementing *Bacillus licheniformis* 809 and *Bacillus subtilis* 810 in a forage-based diet on in situ ruminal disappearance and degradation of DM and fiber fractions of high-concentrate byproduct-based feeds in rumen-cannulated beef cows.

## Materials and methods

This study was conducted at the Montana State University - Bozeman Agricultural Research and Teaching Farm (BART; Bozeman, MT). Experimental procedures involving animals were reviewed and approved by the Montana State University Agriculture Animal Care and Use Committee (protocol #2023-288-AA).

### Animals, experimental design, and diets

Fifteen nonpregnant and rumen-cannulated Angus × Hereford cows [age = 6.1 ± 1 yr; body weight = 655 ± 57 kg] were used in a completely randomized design. Cows were individually housed in 5 × 9 m concrete-floor feeding pens with covered concrete bunks and had free access to grass-mixed hay comprised primarily of Idaho fescue (*Festuca idahoensis*) and Bluebunch wheatgrass (*Pseudoroegneria spicata*), and freshwater. The experimental period lasted 33 d, consisting of 21 d of adaptation (Cappellozza et al. 2012; Limede et al. 2021; Miszura et al. 2023) and 12 d of sample collection as described in the companion manuscript (Limede et al. 2025). Treatments consisted of a forage-based diet plus 500 g of protein-mineral (390 g dried distillers grain + 110 g vitamin-mineral mix; as-fed basis) supplement mixed with **1)** 3 g (as-fed basis) of a *Bacillus*-based DFM containing *Bacillus licheniformis* 809 and *B. subtilis* 810 (**BAC**; *n* = 7; 2.2 × 10^9^ CFU of the mixture/g; Bovacillus, Novonesis, Lyngby, Denmark), or **2)** without BAC (**CON**; *n* = 8). The vitamin-mineral mix consisted of 4.5% Ca, 1.04% P, 3.5% NaCl, 1.02% K, 0.32% Mg, 277 ppm Cu, 1,190 ppm Zn, 4 ppm Se, 459 ppm Mn, 88.18 IU/kg of vitamin A, 8.81 IU/kg of vitamin D3, and 220.4 IU/kg of vitamin E, and 550 mg/kg of monensin (CHS Inc., Sioux Falls, SD). The nutrient profile of the grass-mixed hay and supplements is described in Table 1.

**Table 1:** Nutrient profile of hay and supplement used in this study^1^^,^^2^.

Item	Hay	Supplement^1^	
		CON	BAC
**Nutrient profile, DM basis^2^**			
**DM. %**	93.6	89.2	89.1
**CP, %**	9.1	29.3	29.4
**NDF, %**	63.1	25.9	25.1
**ADF, %**	40.6	10.2	10.8
**CF, %**	2.0	–	–
**Ash, %**	8.96	–	–
**TDN^4^, %**	58	79	79
**ME^5^, Mcal/kg**	2.14	3.34	3.35
**Particle size distribution, %^3^**			
**> 19 mm**	50.2	–	–
**< 19 to > 8 mm**	33.1	–	–
**< 8 to > 4 mm**	3.7	–	–
**< 4 mm**	7.0	–	–

1 Protein supplements were composed of 390 g of dry distillers’ grain plus 110 g of mineral mix.

2 Based on nutritional profile analyzed by a commercial laboratory (Dairy One Forage Laboratory, Ithaca, NY).

3 Penn State Particle Separator procedures (Heinrichs and Knonoff 1996; Kononoff et al. 2003).

4 Calculations were performed according to the equations proposed by (Weiss et al. 1992).

5 Calculations for metabolizable energy used the equations for energy value of feeds described by NASEM (2016).

Throughout the experiment (d 0 to 32), cows were fed daily with chopped grass-mixed hay in an individual concrete feed bunk. The supplements were delivered to each cow at 0800 h on a rubber plate to monitor total consumption before offering hay, as described in Limede et al. (2025). The supplement was totally consumed within 30 min. Daily hay offered was calculated based on the weighted mean intake over the preceding 3 days and was offered at 0830 h, subsequent to complete supplement consumption. Hay intake data are described in the companion manuscript (Limede et al. 2025).

### Ruminal in situ nutrient disappearance and analyses

On day 28 of the experimental period, samples of four (A thorough D; Table 2) different high-concentrate by-product-based diets containing monensin sodium, originated from a commercial feedlot operation in South Africa, were collected and dried in an oven at 55°C for 72 h. Subsequently, the samples were ground on a 2-mm sieve. From days 28 to 32, 4 g of each high-concentrate diet (DM basis) was placed in nylon bags (50 ± 10 μm pore size; 10 × 20 cm; Ankom Tech. Corp., Macedon, NY, USA), introduced through the cannulas, and incubated in the rumen of each cow for 0, 12, 24, 48, 72, and 96 h. Three bags per diet were used at each incubation time. Bags were introduced into the rumen in reverse order so that all bags were removed at 0 h and immediately washed intensively until clear water was obtained. The 0-h bags were included in the final step to assess nutrient loss during washing and were not introduced into the rumen. Once washed, the bags were dried at 55°C for 72 h to calculate the DM content. Sequential detergent fiber analyses were used to determine NDF (Van Soest et al. 1991) and ADF (Goering and Van Soest 1970) with an Ankom 2000 fiber analyzer (Ankom Tech. Corp., Macedon, NY, USA). Sodium sulfite and heat-stable α-amylase were added to the NDF analysis.

**Table 2:** Composition and nutrient profile of the diets used in this study^1^.

Item	Diet A	Diet B	Diet C	Diet D
***Composition, % DM***				
** Hominy chop**	57.0	62.0	67.0	72.0
** Eragrostis hay**	24.0	19.0	14.0	9.0
** Molasses**	7.0	7.0	7.0	7.0
** Corn silage**	6.0	6.0	6.0	6.0
** Cottonseed cake**	2.8	2.8	2.8	2.8
** Limestone**	1.6	1.6	1.6	1.6
** Urea**	1.1	1.1	1.1	1.1
** Salt**	0.4	0.4	0.4	0.4
** Mineral-vitamin premix**	0.1	0.1	0.1	0.1
***Nutritional profile***				
** DM, %**	89.4	90.3	89.7	89.8
** CP, %**	15.0	14.6	13.9	12.6
** NDF, %**	29.6	27.2	23.8	19.3
** ADF, %**	16.6	14.6	15.5	7.5
** Hemicellulose, %**	13.0	12.6	11.9	11.8
** TDN^2^, %**	72.0	73.0	72.0	76.0
** NE_m_^3^, Mcal/kg**	1.74	1.76	1.78	1.84
** NE_g_^3^, Mcal/kg**	1.12	1.14	1.14	1.21

1 The diets used in this trial reflect a typical progressive step-up program commonly implemented in South African feedlot systems to transition cattle from a forage-based to a high-concentrate by-product-based regimen. All diets were sent to a commercial laboratory (Dairy One Forage Laboratory, Ithaca, NY) for determination of the nutritional profile.

2 Calculations were performed according to the equations proposed by (Weiss et al. 1992).

3 Calculations for net energy for maintenance and gain used the equations for energy value of feeds described by NASEM (2016).

The high-concentrate by-product-based feedlot diets represented those often employed in South African dietary management and consisted of hominy chop, corn silage, eragrostis hay, cottonseed cake, molasses syrup, urea, limestone, salt, and a commercial mineral-vitamin premix (Table 2). The diets were labeled from A through D and sent to a commercial laboratory (Dairy One Forage Laboratory, Ithaca, NY) for determination of the nutritional profile (Table 2). It is important to note that a grass-hay basal diet was used to maintain a stable and consistent ruminal environment, minimizing variation in pH and digesta passage rate. This created a controlled system for assessing the effects of *Bacillus* supplementation on the in situ degradation of high-concentrate, by-product-based feedlot diets. Using a forage-based baseline allowed us to isolate microbial activity attributable specifically to the DFM, ensuring that observed responses reflected the activity of *Bacillus* spp. rather than diet-induced ruminal fluctuations. Although this approach provided a consistent and controlled platform for comparison, it does not perfectly mimic the ruminal conditions of cattle fed high-concentrate feedlot diets. Even so, under these standardized conditions, the in situ degradation of high-concentrate by-product feeds could be evaluated reliably.

### Statistical analysis

Data were analyzed using a completely randomized design with cow as the experimental unit and the MIXED procedure of SAS version 9.4 (SAS Inst. Inc., Cary, NC). Satterthwaite approximation was used to determine the denominator degrees of freedom for the test of fixed effects, and cow (treatment) as a random variable. The model statement for the disappearance of DM, NDF, and ADF included the effects of treatment, hour, and the treatment × hour interaction. The specified term for these repeated statements was hour, with cow (treatment) as the subject, and autoregressive as the covariance structure based on the Akaike information criterion. Kinetic parameters of forage DM, NDF disappearance were estimated using nonlinear regression procedures of SAS (SAS Inst. Inc., Cary, NC), as described by Vendramini et al. (2008). Effective degradability of forage DM and NDF was calculated by fixing ruminal passage rate at 0.046/h (Poore et al. 1990), and using the model proposed by Ørskov and McDonald (1979) and adjusted by Fadel (1992; 2004), whereas treatment effects on these parameters were analyzed using the MIXED procedure of SAS (SAS Inst. Inc.). For all analyses performed, significance was set at *P* ≤ 0.05, and tendencies were noted if 0.05 < *P* ≤ 0.10.

## Results and discussion

The diets used in this trial reflect a typical progressive step-up program commonly implemented in South African feedlot systems to transition cattle from a forage-based to a high-concentrate by-product-based regimen. Four commercial diets (A-D) were formulated with incremental changes in nutrient composition to promote ruminal adaptation and reduce the risk of digestive disturbances. The DM content remained consistently high (89.4-90.3%), while CP ranged from 15% in diet A to 12.6% in diet D. Fiber fractions exhibited substantial reductions across diets: NDF decreased from 29.6% in diet A to 19.3% in diet D, and ADF ranged from 16.6% to 7.5%, reflecting the gradual reduction in roughage inclusion, mainly eragrostis hay. Hemicellulose content followed a similar trend (13.0 to 11.8%), supporting the shift toward more digestible ingredients. The TDN increased slightly (72.0 to 76.0%), accompanied by incremental improvements in NEm (1.74 to 1.84 Mcal/kg) and NEg (1.12 to 1.21 Mcal/kg), consistent with the energy-dense nature of finishing diets.

No treatment × hour interactions were detected (*P* ≥ 0.11) for nutrient disappearance in any of the diets (Table 3); therefore, only the main treatment effects were reported throughout the manuscript. Dry matter and NDF disappearance were greater (*P* ≤ 0.05) in diets A, C, and D for cows fed BAC compared with CON, whereas no differences were observed in nutrient disappearance from diet B (*P* ≥ 0.33). Similarly, ADF disappearance was greater in diet A (*P* = 0.04), tended to be greater in diet C (*P* = 0.07), and was unaffected by treatment in diets B and D (*P* ≥ 0.13). In the companion manuscript, Limede et al. (2025) reported that the supplementation of *Bacillus licheniformis* 809 and *B. subtilis* 810 to rumen-cannulated beef cows enhanced voluntary forage DMI, optimized ruminal in situ disappearance of forage DM and NDF, increased apparent total-tract digestibility of DM, CP, NDF, and ADF, and rumen pH post-feeding. However, it is noteworthy mentioning that these greater nutrient disappearance and, therefore, nutrients available in the rumen did not result in alterations to the rumen bacterial community of the rumen-cannulated beef cows, as no significant changes were observed on the molar proportions of short-chain fatty acids (**SCFA**) and the main phyla, families, and diversity indices following BAC supplementation (Limede et al. 2025). Based on these results, the aforementioned effects may be related to the ability of *Bacillus spp.* to adhere to feed particles and secrete enzymes (Schallmey et al. 2004; Luise et al. 2022).

**Table 3:** In Situ nutrient disappearance (%) of high-concentrate by-product-based diets (A through D) incubated in rumen-cannulated beef cows fed forage-based diets and supplemented or not (**CON**; *n* = 8) with a *bacillus*-based direct-fed microbial (**BAC**; *n* = 7)^1^^,^^2^.

Item	Treatments		SEM	*P*-value^3^		
	CON	BAC		T	H	T × H
***Diet A***						
** DM, %**	75.0	78.0	0.77	0.02	< 0.0001	0.47
** NDF, %**	41.9	47.0	1.72	0.05	< 0.0001	0.99
** ADF, %**	22.9	29.7	2.18	0.04	< 0.0001	0.94
***Diet B***						
** DM, %**	74.8	77.2	1.64	0.34	< 0.0001	0.61
** NDF, %**	37.8	42.9	3.38	0.33	< 0.0001	0.52
** ADF, %**	21.6	27.5	3.88	0.32	< 0.0001	0.24
***Diet C***						
** DM, %**	79.8	83.1	0.79	0.01	< 0.0001	0.11
** NDF, %**	40.8	50.6	2.41	0.02	< 0.0001	0.18
** ADF, %**	23.1	26.9	2.45	0.09	< 0.0001	0.51
***Diet D***						
** DM, %**	79.3	82.1	0.88	0.05	< 0.0001	0.45
** NDF, %**	41.2	50.4	2.19	0.02	< 0.0001	0.35
** ADF, %**	23.7	31.8	3.56	0.13	< 0.0001	0.12

1 Bags were incubated at 0, 12, 24, 48, 72, and 96 h in the rumen of the cows.

2 Treatments were mixed into 500 g of protein-mineral (390 g dry distillers’ grain + 110 g mineral mix; as-fed basis).

3 T = treatment effect; H = hour effect; T × H = treatment × hour interaction.

In the current study, DM and NDF disappearance were greater in BAC-fed cows in 3 of the 4 high-concentrate diets evaluated, whereas ADF disappearance was greater in 2 of the 4 diets. Improvements in DM and NDF disappearance in BAC-fed cows ranged from 3.5 to 4.0% and 12 to 23%, respectively. For the ADF disappearance, the improvements in diets A and C ranged from 16% to 29%. Previously, in vitro studies also reported greater DM and NDF disappearance following BAC incubation with forage-based substrates (Pan et al. 2022; Cappellozza et al. 2023b) or with dairy TMR (Cappellozza et al. 2023b). In situ degradation provides a practical measure of how feed components are broken down within the rumen and serves as a proxy for their potential contribution to nutrient availability in vivo (Foster et al. 2023). This information is critical because greater ruminal degradation generally improves energy supply and supports microbial growth, which are essential for sustaining DMI and optimizing animal performance. For instance, beef steers offered a forage-based diet supplement with ground barley, total-tract nutrient digestibility (DM and NDF) increased following BAC supplementation, but no treatment effects were observed when a high-concentrate diet was fed (Eyre et al. 2025). In dairy cattle, in situ DM disappearance and apparent total-tract DM digestibility were increased by adding BAC to a corn silage-based TMR diet (Arthur et al. 2025), while similar results on DM digestibility tended to be observed in a TMR containing 28.5% starch (Terré et al. 2024). Hence, it is feasible to speculate that BAC supplementation has been promoting nutrient degradation of feedstuffs and/or TMR with different nutritional profiles, but there is still missing information regarding the fractions of nutrients being potentially impacted. It is important to note that this study evaluated whether in situ ruminal nutrient disappearance would be similarly affected when a diet with different nutritional characteristics (high-concentrate byproduct-based) was incubated in cows consuming a forage-based diet supplemented with *B. licheniformis* 809 and *B. subtilis* 810, likely reflecting a distinct basal rumen microbiota profile. We acknowledge, however, that the forage-based model limits inference regarding whether Bacillus-DFM supplementation would produce comparable responses in animals fed high-concentrate diets. Ruminal conditions under grass hay differ from concentrate feeding in pH dynamics, fermentation patterns, and microbial community structure and these factors could influence the efficacy or mechanism of action of the DFM. Our intent in using a grass-hay basal diet was to provide a controlled and stable environment for in situ comparisons; nonetheless, this approach does not replicate the rumen environment of typical feedlot diets. Therefore, our assumptions are restricted to the effects observed under a forage-based baseline, and future studies should evaluate Bacillus-DFM directly in cattle consuming high-concentrate diets to determine whether the response is diet-dependent.

When the four feedlot diets were pooled for further analysis, the fraction B (degradable fraction; total pool disappearing at a measurable rate) of DM was greater for BAC compared with CON (*P* = 0.02), whereas the fraction C (undegradable fraction; total unavailable pool in the rumen) tended to be lower for BAC than CON (*P* = 0.08; Table 4). No further treatment effects were observed on DM fractional degradation rates (% per h; *P* = 0.97) or effective degradability (*P* = 0.11; Table 4) between treatments. Evaluating combined NDF degradation parameters across diets, the fraction B tended to be greater for BAC (*P* = 0.10), whereas effective degradability was greater for BAC compared with CON (*P* = 0.02; Table 4). No treatment effects were observed on NDF fraction C (*P* = 0.18; Table 4) or degradation rates (% per h; *P* = 0.92) between treatments. Based on the results of the current study, BAC supplementation increased the pool of the potentially degradable fraction (fraction B) of DM and showed a tendency to improve the same pool in NDF. Compared with CON cohorts, BAC-fed cows exhibited 11 and 12% greater degradation of fraction B values for DM and NDF, respectively. This improvement in the potentially degradable fraction of diets might be partially explained by the ability of *B. subtilis* to produce and release expansin-like proteins (Pech-Cervantes et al. 2019), which can loosen, expand, or disrupt plant cell wall components, such as cellulose and hemicellulose (Liu et al. 2015), yielding better nutrient degradation and utilization. Accordingly, in dairy cow TMR with NDF values varying from 25 to 34%, BAC increased in vitro NDF degradation by 11 and 12% at 24 and 48 h, respectively (Cappellozza et al. 2023b). Effective DM and NDF degradability were greater in beef heifers fed a low-quality tropical grass and supplemented with 1.2 kg of a corn-based supplement supplemented with BAC (De Souza et al. 2026). Moreover, Bunterngsook et al. (2014) observed greater synergism between expansin-like proteins and fibrolytic enzymes in substrates containing a higher proportion of structural carbohydrates, likely due to a more effective breakage of hydrogen bonds between hemicellulose and cellulose, thereby increasing accessibility of cellulases to cell wall polysaccharides (Saloheimo et al. 2002). Moreover, rumen pH did not limit microbial activity or function (Limede et al. 2025), allowing effective fermentation of structural carbohydrates (NASEM 2016). Therefore, the enhanced nutrient degradability observed under varying dietary conditions appears to be driven by enzyme production from *B. licheniformis* 809 and *B. subtilis* 810.

**Table 4:** Pooled fractional degradation of dry matter and neutral detergent fiber of high-concentrate by-product-based diets (A through D) incubated in the rumen of rumen-cannulated beef cows fed forage-based diets and supplemented or not (**CON**; *n* = 8) with a *Bacillus*-based direct-fed microbial (**BAC**; *n* = 7)^1^^,^^2^.

Degradation Parameters^3^	Treatments		SEM	*P*-value
	CON	BAC		
**DM**				
** Fractions, %**				
** A**	47.5	45.7	0.95	0.19
** B**	37.7	41.9	1.20	0.02
** C**	14.8	12.3	0.95	0.08
** Kd, %/h^4^**	4.25	4.27	0.295	0.97
** Effective degradability, %^5^**	72.1	74.3	0.94	0.11
**NDF**				
** Fractions, %**				
** A**	14.8	13.1	1.61	0.47
** B**	52.3	58.9	2.80	0.10
** C**	32.9	28.0	2.53	0.18
** Kd, %/h^4^**	2.96	3.03	0.55	0.92
** Effective degradability, %^5^**	39.4	45.4	1.64	0.02

1 Bags were incubated at 0, 12, 24, 48, 72, and 96 h in the rumen of the cows.

2 Treatments were mixed into 500 g of protein-mineral (390 g dry distillers’ grain + 110 g mineral mix; as-fed basis).

3 A = soluble fraction (total pool disappearing at a rate too rapid to measure); B = degradable fraction (total pool disappearing at a measurable rate); C = undegradable fraction (total pool unavailable in the rumen).

4 Fractional rate constant (% per h).

5 Calculated by fixing ruminal passage rate at 0.046/h (Poore et al. 1990) and using the model proposed by Ørskov and McDonald (1979) and adjusted by Fadel (1992, 2004).

A novel finding of the present study was that improvements in nutrient disappearance and degradation fractions were observed even though the forage-based diets fed to the animals differed markedly from the high-concentrate diets evaluated in the in situ bags. This suggests that the treatments influenced ruminal dynamics in a way that enhanced the breakdown of feed components, regardless of the compositional contrast between the basal diet offered to the donor cows (forage-based) and the incubated substrates (high-concentrate byproducts-based diets). Such an outcome highlights the potential for dietary strategies, such as BAC, to modulate ruminal degradability beyond the immediate characteristics of the diet, offering practical implications for improving nutrient utilization efficiency across diverse feeding systems. An important question is whether BAC supplementation influences ruminal degradability and digestibility through changes in microbial composition or by other mechanisms. Across treatments, the ruminal microbiome remained dominated by *Firmicutes, Bacteroidota*, *Actinobacteria*, and *Patescibacteria*, together accounting for approximately 96% of identified phyla (Limede et al. 2025). The most abundant families included *Lachnospiraceae*, *Prevotellaceae*, *Christensellaceae*, *Oscillospiraceae*, *Rikenellaceae*, and *Ruminococcaceae* (68% of families; Limede et al. 2025). *Lachnospiraceae* and *Ruminococcaceae* are key cellulolytic groups linked to fiber degradation and butyrate production, while Prevotella contributes to succinate and acetate synthesis (Hernandez-Sanabria et al. 2010; Ramakrishna 2013; Meehan and Beiko 2014; Nograšek et al. 2015; La Reau and Suen 2018). Similar microbial profiles have been reported in studies using high-concentrate diets (Pickett et al. 2022; Linde et al. 2023), reinforcing the strong influence of diet on ruminal community structure. Notably, BAC improved nutrient disappearance and degradation fractions without major shifts in bacterial composition, supporting the hypothesis that its effects are driven by enhanced enzymatic activity rather than microbial restructuring (Limede et al. 2025).

Recently, Detmann et al. (2024) reviewed how NDF degradation of tropical pastures impacts forage voluntary DMI and performance of grazing beef cattle. For high-concentrate diets, other authors have demonstrated the importance of maximizing nutrient degradation in the rumen, and focus has been given on starch (Vander Pol et al. 2008; Owens et al. 2016). Nonetheless, the forage portion of the diet, and therefore the fiber fractions, is essential to maintain the health status of the rumen (Owens et al. 1998) and to stimulate nutrient intake (Galyean and Defoor 2003; Arelovich et al. 2008). Marques et al. (2016) reported that the inclusion of a low-quality roughage (sugarcane bagasse) in high-concentrate diets increased feed intake and average daily gain, with a tendency to improve carcass traits in *Bos indicus* feedlot cattle, highlighting the importance of stimulating fibrous nutrient utilization. In *B. indicus* bulls, Dias et al. (2022) observed improved feed efficiency and positive alterations in rumen fermentation when BAC was included in a byproduct-based diet containing 42% NDF and 33% starch. Similarly, Cordeiro et al. (2024) reported reduced DMI and fecal starch, but enhanced feed efficiency in crossbred beef bulls fed BAC in a high-starch feedlot diet (22% NDF and 50% starch) over a 100-day period. Collectively, these findings indicate that BAC supplementation may enhance feed efficiency and nutrient utilization across varied diet compositions by increasing bacterial adhesion to feed particles and promoting the secretion of enzymes that degrade dietary nutrient components ((Schallmey et al. 2004; Luise et al. 2022).).

## Conclusion

In summary, supplementation with a Bacillus‑based DFM containing B. licheniformis 809 and *B. subtilis* 810 enhanced the in situ disappearance of DM, NDF, and ADF in high‑concentrate byproduct‑based diets and increased the pool of potentially degradable material, even when evaluated under a controlled forage‑based system designed to isolate microbial effects attributable specifically to the DFM. Although this controlled approach does not fully replicate the rumen environment of cattle fed high‑concentrate diets, it allowed reliable assessment of *Bacillus*‑driven degradation responses, underscoring the need for further research to determine their impacts on total‑tract digestibility, rumen function, microbiota, and feedlot performance.

## CRediT statement

Arnaldo C. Limede (Investigation, Methodology, Writing - original draft), Rodrigo S. Marques (Methodology, Supervision, Validation, Writing - review & editing), Fernando A. A. Cidrini (Methodology, Writing - original draft), Erollykens F. Santos (Methodology, Writing - original draft), Paolo Fantinati (Investigation, Methodology), Bruno I. Cappellozza (Investigation, Methodology, Writing - original draft, Writing - review & editing).

## List of Abbreviations

ADF: Acid detergent fiber
CFU: Colony forming unit
DFM: Direct-fed microbials
DM: dry matter
DMI: dry matter intake
GIT: gastrointestinal tract
NDF: neutral detergent fiber
SCFA: Short-chain fatty acids
TMR: total mixed ration
